# Vibration Noise Modeling for Measurement While Drilling System Based on FOGs

**DOI:** 10.3390/s17102367

**Published:** 2017-10-17

**Authors:** Chunxi Zhang, Lu Wang, Shuang Gao, Tie Lin, Xianmu Li

**Affiliations:** Key Laboratory of Inertial Technology, Institute of Opto-electronics Technology, School of Instrument Science and Opto-electronics Engineering, Beihang University, Beijing 100191, China; zhangchunxi@buaa.edu.cn (C.Z.);wanglu@buaa.edu.cn (L.W.); opticlin@163.com (T.L.); lixianmu@buaa.edu.cn (X.L.)

**Keywords:** FOG-based MWD, system noise model, vibration noise, dynamic Allan variance

## Abstract

Aiming to improve survey accuracy of Measurement While Drilling (MWD) based on Fiber Optic Gyroscopes (FOGs) in the long period, the external aiding sources are fused into the inertial navigation by the Kalman filter (KF) method. The KF method needs to model the inertial sensors’ noise as the system noise model. The system noise is modeled as white Gaussian noise conventionally. However, because of the vibration while drilling, the noise in gyros isn’t white Gaussian noise any more. Moreover, an incorrect noise model will degrade the accuracy of KF. This paper developed a new approach for noise modeling on the basis of dynamic Allan variance (DAVAR). In contrast to conventional white noise models, the new noise model contains both the white noise and the color noise. With this new noise model, the KF for the MWD was designed. Finally, two vibration experiments have been performed. Experimental results showed that the proposed vibration noise modeling approach significantly improved the estimated accuracies of the inertial sensor drifts. Compared the navigation results based on different noise model, with the DAVAR noise model, the position error and the toolface angle error are reduced more than 90%. The velocity error is reduced more than 65%. The azimuth error is reduced more than 50%.

## 1. Introduction

In the oil industry, horizontal drilling processes make use of Measurement While Drilling (MWD) instruments to monitor the position and the orientation of the bottom hole assembly (BHA). The traditional MWD instrument is comprised of three accelerometers and three magnetometers. The magnetometers determine the orientations by measuring the earth magic field. The major defect using the magnetometers is that their accuracy is influenced by the magnetic field existing in the oil hole. Consequently, the deteriorative measurements accuracy could lead to drilling failure [1]. For the sake of improving the survey accuracy, gyroscopic measure techniques based on fiber optic gyroscopes (FOGs) have been put forward to replace the magnetic instruments [2]. Though the FOG-based inertial navigation technique is a mature technique in the military domain, it encounters a lot of special problems because of the harsh work environment of drilling. It needs to work over 200 h with a high shock and strong vibration [1]. As we all know, the inertial errors accumulate with time, which leads to accuracy degradation. For long-term measuring and high accuracy surveying, the external aiding observations are fused into the FOG-based MWD by Kalman filter (KF), such as the drilling pipe length, the penetration rate and the zero velocity update (ZUPT) [3,4,5]. In KF, the inertial sensor’s noise should be modeled to be used as the system noise. They were often modeled as the stationary white Gaussian noise. However, during the vibration, the gyroscope noise is no longer stationary white Gaussian noise. What’s worse, an incorrect system noise model will influence the performance and accuracy of KF greatly. Therefore, it is very critical to put forward an accurate modeling approach for the system noise.

Various approaches were researched to model the random errors in the gyros and accelerometers. In Reference [6], the stochastic errors in the gyroscope were modeled with the autoregressive moving average model (ARMA). In Reference [7], the stochastic errors in gyros and accelerometers were simplified as an autoregressive (AR) model. But the ARMA model and the AR model were unsuitable to process higher-order stochastic processes, and high-dynamic ranges [8]. In Reference [9], the stochastic errors were assumed to be a stationary Gaussian-Markov process. Reference [10] pointed out that the stochastic noise of the inertial sensors were not stationary. Thus, the stationary Gaussian-Markov process was not an accurate model for the stochastic process. What’s worse, it is very difficult to determine the order, the coefficients, and the time constant of a Gaussian-Markov model for a particular stochastic noise. In Reference [11], the stochastic errors in the inertial sensors were assumed to be a white noise process. The user’s manual or data sheet of inertial sensors often disturb the power spectral density functions (PSDs) of this white noise process. Based on the PSD, the noises covariance matrix (Q) of the white noise which was needed in the Kalman Filter could be obtained. However, the real stochastic process in an inertial sensor may be much more complicated than white noise. For most applications this assumption is an approximation and a simple model; it sacrifices performances and precision. Reference [12] designed a colored-noise model for KF to diminish the effects of the vibration error. But this method was verified by the simulation. In reference [13], the random noise of the Micro-Electro-Mechanical System (MEMS) inertial sensor were identified and modeled by Allan variance. This model was applied into this low cost Inertial navigation system (INS) integrated system to improve the accuracy and performance of the system. But in order to simplify the model, some components of the stochastic noise were ignored, such as Quantization noise. In References [14,15], the stochastic errors in an inertial sensor were identified by Allan variance, and an equivalent differential equation representation for each kind of stochastic noise was established. The equivalent differential equation was augmented into the KF of GPS/INS integration. However, after augmenting, the KF estimation process will become much more complicated and take more time.

In 2003, L. Galleani and P. Tavella developed the dynamic Allan variance (DAVAR) to track and reveal the anomy and non-stationary in the atom clock behavior [16]. In contrast to Allan variance, DAVAR can track and reveal the non-stationary characteristics of time series [17]. Li et al. [18], Wei et al. [19] and Zhang et al. [20] utilized the DAVAR to describe the non-stationary of the laser gyroscope. Wang et al. took advantage of DAVAR to identify and characterize the vibration noise of FOGs in the MWD system [21,22]. However, DAVAR hasn’t been applied to model the random noise to improve the performance of the KF. In this paper, the gyroscope vibration noise is identified by DAVAR, and then a noise model based on DAVAR was developed for the first time. With this accurate vibration noise model, the performance and the accuracy of the KF could be improved.

The organization of this paper is as follows. In the Section 2, the Dynamic Allan variance method is introduced simply. Section 3, the KF for MWD is designed. Section 4, the new noise modeling method was developed and the KF based on the new model is designed. The experimental results are presented in the Section 5. Section 6 is the conclusion.

## 2. Dynamic Allan Variance

The Allan variance for FOGs is defined as follows [23],

(1) $$\sigma_{\omega }^{2}(\tau )=\frac{1}{2}\langle {\left(\bar{\omega }(t+\tau )-\bar{\omega }(t)\right)}^{2}\rangle$$

where τ is the observation interval, ω(t) is FOG output data. and 〈〉 is a symbol indicating a time averaging. The average of $\bar{\omega }(t)$ is calculated by

(2) $$\bar{\omega }(t)=\frac{1}{\tau }\int_{t}^{t+\tau }\omega (u)du$$

where u is the integral variable.

The dynamic Allan variance (DAVAR) is developed based on Allan variance. It is a sliding Allan variance. Its computation process can be described as following. Firstly, at a given time epoch, we truncate the FOG data with a rectangular window. Secondly, the Allan variance of the truncated data could be calculated using Equation (1). As a result, we get the Allan variance at a given time epoch. Then repeating these two steps at every time epoch, the Allan variance at each time epoch can be obtained. In the end, plotting all the variances in a 3D graph, we can obtain the DAVAR figure. Please refer to reference [17] to get the detailed computation process. In this paper, we describe the definition of DAVAR as shown in Equation (3).

(3) $$\sigma_{\omega }^{2}(t,\tau )=\frac{1}{2\tau^{2}(N_{w}-2\tau )}\int_{t-\frac{N_{w}}{2}+\tau }^{t+\frac{N_{w}}{2}-\tau }\left(\bar{\omega }(u+\tau )-\bar{\omega }(u\right){)}^{2}du$$

where $N_{w}$ is the length of the truncation window, *t* is the analysis time epoch, τ is the observation interval, $\sigma_{\omega }^{2}(t,\tau )$ is the DAVAR.

## 3. Noise Modeling Based on DAVAR

El-Sheimy et al. [24] showed that a unique relationship existing between Allan variance $\sigma_{\omega }^{2}(\tau )$ and the power spectral density (PSD) of the random noise. It is,

(4) $$\sigma_{\omega }^{2}(\tau )=4\int_{0}^{\infty }S_{\omega }(f)\frac{\sin^{4}(\pi f\tau )}{{\left(\pi f\tau \right)}^{2}}du$$

where $S_{\omega }(f)$ is the PSD of the random process ω(t). Substituting PSD of any physical meaningful random process into Equation (4,), we can obtain the Allan variance $\sigma_{\omega }^{2}(\tau )$ of this random process [23]. As far as we all know, there are five kinds of noise existing in the random noise of the gyros. These are the quantization noise (*Q*), angular random walk (*N*), bias instability (*B*), rate random walk (*R*), and the rate slope (*K*). The quantization noise is one of the errors introduced into an analog signal by encoding it in digital form. The gyro angle random walk was contributed by the high-frequency noise terms that have correlation time much shorter than the sample time. They come from the light path noise of the gyro. The origin of bias instability noise is the electronics or other components that are susceptible to random flickering. Because of its low-frequency nature, it is indicated as the bias fluctuations in the data. Rate random walk noise is a random process of uncertain origin, possibly a limiting case of an exponentially correlated noise with a very long correlation time. The rate slope is considered to be a kind of deterministic error. It is caused by the slowly changing of the light source intensity or caused by the temperature of the environment.

When the PSD of one random process passed through a filter with the transfer function $\frac{\sin^{4}(\pi f\tau )}{{\left(\pi f\tau \right)}^{2}}$, we can get its corresponding Allan variance. That means that different types of random processes can appear in different region of τ and the different types of random noise can be examined by regulating τ [25]. With this particular characteristic, the various noise terms existing in the gyro could be identified. Table 1 summarized a relationship between noise terms and the Allan variance and observation τ.

Assuming that the noise terms existing in the gyro are statistically independent, the Allan variance could be rewritten as the sum of the Allan variances due to each random process at the different τ [25]. In other words,

(5) $$\begin{array}{ccc} \sigma^{2}(\tau ) & = & \sigma_{Q}^{2}(\tau_{Q})+\sigma_{N}^{2}(\tau_{N})+\sigma_{B}^{2}(\tau_{B})+\sigma_{K}^{2}(\tau_{K})+\sigma_{R}^{2}(\tau_{R})\\ & = & \frac{3Q^{2}}{\tau_{Q}{}^{2}}+\frac{N^{2}}{\tau_{N}}+\frac{2B^{2}}{\pi }\ln2+\frac{K^{2}\tau_{K}}{3}+\frac{R^{2}\tau_{R}{}^{2}}{2}\\ & = & C_{-2}\tau_{Q}{}^{-2}+C_{-1}\tau_{N}{}^{-1}+C_{0}\tau_{B}{}^{0}+C_{1}\tau_{K}{}^{1}+C_{2}\tau_{R}{}^{2} \end{array}$$

where $\sigma_{Q}^{2}(\tau_{Q}),\sigma_{N}^{2}(\tau_{N}),\sigma_{B}^{2}(\tau_{B}),\sigma_{K}^{2}(\tau_{K}),\sigma_{R}^{2}(\tau_{R})$ stand for Allan variance of each random noise term. $\tau_{Q},\tau_{N},\tau_{B},\tau_{K}$, and $\tau_{R}$ are the observation times of individual random processes, respectively. $C_{-2},C_{-1},C_{0},C_{1}$ and $C_{2}$ are the polynomial coefficients of the $\sigma^{2}(\tau )-\tau$. With the line fitting approach, the relationship between the noise coefficients and the coefficients of the $\sigma^{2}(\tau )$ could be established. If the unit of gyroscope signal is degree per hour (°/h), each noise coefficient could be obtained as follows:

(6) $$N=\frac{\sqrt{C_{-1}}}{60}({}^{\circ}/h^{\frac{1}{2}})，K=60\sqrt{3C_{1}}({}^{\circ}/h^{\frac{3}{2}})，B=\frac{\sqrt{C_{0}}}{0.664}({}^{\circ}/h)，Q=\frac{10^{6}\pi \sqrt{C_{-2}}}{180\times 3600\times \sqrt{3}}(\prime \prime )，R=3600\sqrt{2C_{2}}({}^{\circ}/h^{2})$$

DAVAR is an assembling of the Allan variances at each the time point. Therefore, at any given time epoch, the DAVAR can be rewritten as Equation (7):

(7) $$\sigma^{2}(t,\tau )=\sigma_{Q}^{2}(t,\tau_{Q})+\sigma_{N}^{2}(t,\tau_{N})+\sigma_{B}^{2}(t,\tau_{B})+\sigma_{K}^{2}(t,\tau_{K})+\sigma_{R}^{2}(t,\tau_{R})$$

Fitting the σ(t,τ)−τ curve, the noise coefficients *Q*(*t*), *N*(*t*), *B*(*t*), *K*(*t*), and *R*(*t*) at that time *t* could be obtained. Therefore, coefficients for all the time *t* could be obtained. With all these coefficients, we can establish the dynamic model for the random error of the gyroscope.

Institute of Electrical and Electronics Engineers (IEEE) 952 1997 [23] puts forward that the magnitude of the five noise term can be read off from the slope line of the log σ verse log τ curve, namely bi-logarithmic curves. The magnitude of Quantization noise can be read off from the logσ–logτ curve at $\tau_{Q}\;=\;\sqrt{3}$, the magnitude of Angle random walk noise can be read off from the bi-logarithmic curves of slop −0.5 at $\tau_{N}\;=\;1$. The magnitude of Rate random walk can be obtained from the bi-logarithmic curves at $\tau_{R}\;=\;\sqrt{2}$ and the magnitude of the Rate Slope noise can be read off at $\tau_{K}\;=\;3$. The numerical value of the bias instability has nothing with τ. Therefore, for a given time *t*, substituting observation time $\tau_{Q}$, $\tau_{N}$, $\tau_{R}$, $\tau_{K}$ and their corresponding noise coefficients into Equation (5), we can obtain the accurate Allan variance including all kinds of noise. This accurate Allan variance which changes with time can be described as follows

(8) $$\begin{array}{ccc} \sigma^{2}(t) & = & \sigma_{Q}^{2}(t,\tau_{Q})+\sigma_{N}^{2}(t,\tau_{N})+\sigma_{B}^{2}(t)+\sigma_{K}^{2}(t,\tau_{K})+\sigma_{R}^{2}(t,\tau_{R})\\ & = & \frac{3Q^{2}}{\tau_{Q}^{2}}+\frac{N^{2}}{\tau_{N}^{2}}+\frac{2B^{2}}{\pi }\ln2+\frac{K^{2}\tau_{K}}{3}+\frac{R^{2}\tau_{R}^{2}}{2} \end{array}$$

$\sigma^{2}(t)$ in Equation (8) is the accurate noise model which could track and exhibit the non-stationary characteristics of the noise. What’s more, it not only contains the white noise, such as angler random walk, but also the color noise, such as Quantization noise, Bias instability noise, Rate random walk noise and Rate lamp noise. Applying this noise model to the Kalman filter may improve the performance of the Kalman filter greatly, which we will discuss in the next section.

## 4. DAVAR Aided Kalman Filter

There are two types of continuous aiding observations which can be fused into the MWD surveying system [1]. The first one is the position information that can be obtained from the continuous measurement of the drill pipe length. The second one is the velocity which can be derived from pipe length and time. Therefore, position/velocity loose coupled navigation approach for MWDs is chosen in this paper.

### 4.1. Kalman Filter Model

The state equation is expressed as follows:

(9) $$X_{k}=F_{k,k-1}X_{k-1}+G_{k-1}W_{k-1}$$

(10) $$X_{k-1}={\left[\begin{array}{cccccccccccc} \delta L & \delta \lambda & \delta h & \delta V^{e} & \delta V^{n} & \delta V^{u} & \delta I & \delta T & \delta A & aB_{x} & aB_{y} & \begin{array}{cccc} aB_{z} & gB_{x} & gB_{y} & gB_{z} \end{array} \end{array}\;\right]}^{T}$$

(11) $$W_{k-1}={\left[0_{1\times 3}\;w_{a}^{T}\;w_{g}^{T}\;0_{1\times 6}\right]}^{T}$$

where $X_{k}$ is the error states vector. δL is the latitude error, δλ is longitude error, and δh are height error. $\delta V^{e}$ is the east velocity errors, $\delta V^{n}$ is the north velocity error, and $\delta V^{u}$ is up velocity error. δI is the inclination error, δT is the toolface angle error and δA is azimuth error. $aB_{x}$, $aB_{y}$ and $aB_{z}$ are the bias errors of accelerometers along the *X* axis, *Y* axis and *Z* axis, respectively. $gB_{x}$, $gB_{y}$ and $gB_{z}$ are the bias errors of gyroscope along with the *X* axis, *Y* axis and *Z* axis, respectively. $F_{k,k-1}$ is the dynamic matrix relating $X_{k-1}$ to $X_{k}$. $G_{k-1}$ is the noise coefficient matrix, and $W_{k-1}$ is the system noise vector which has the normal distribution with the variance matrix $Q_{k-1}$. $w_{a}$ is the noise model of accelerometers and $w_{g}$ is the noise model of gyroscopes.

The measurement equation is

(12) $$Z_{k}=H_{k}\cdot X_{k}+\;V_{k}$$

(13) $$Z_{k}={\left[\begin{array}{cccccc} L_{ins}-\;L_{update} & \lambda_{ins}-\;\lambda_{update} & h_{ins}-\;h_{update} & V_{ins}^{e}-V_{update}^{e} & V_{ins}^{n}-V_{update}^{n} & V_{ins}^{u} \end{array}-V_{update}^{u}\;\right]}^{T}$$

$Z_{k}$ is external measurements or observations, $H_{k}$ is the observation matrix. $V_{k}$ is the random noise vector for the observations. $V_{k}$ is random noise model of the observations. ${\left[\begin{array}{ccc} V_{ins}^{e} & V_{ins}^{n} & V_{ins}^{u} \end{array}\right]}^{T}$ is the velocity obtained from the INS. ${\left[\begin{array}{ccc} V_{update}^{e} & V_{update}^{n} & V_{update}^{u} \end{array}\right]}^{T}$ is the drill bit rate of penetration. ${\left[\begin{array}{ccc} L_{ins} & \lambda_{ins} & h_{ins} \end{array}\right]}^{T}$ is the position calculated by the INS. ${\left[\begin{array}{ccc} L_{update} & \lambda_{update} & h_{update} \end{array}\right]}^{T}$ is the position calculated by the drilling pipe length.

Traditionally, the observation random noise vector $V_{k}$ and the system measurement noise $W_{k}$ are both assumed to be white sequence and not correlated with each other. The characteristics and relationship of $W_{k}$ and $V_{k}$ are expressed as

(14) $$E\left[W_{k}\right]=0,Cov\left[W_{k},W_{j}\right]=E\left[W_{k}W_{j}{}^{T}\right]=Q_{k}\delta_{kj}$$

(15) $$E\left[V_{k}\right]=0,Cov\left[V_{k},V_{j}\right]=E\left[V_{k}V_{j}{}^{T}\right]=R_{k}\delta_{kj}$$

(16) $$Cov\left[W_{k},V_{j}\right]=E\left[W_{k}V_{j}{}^{T}\right]=0$$

(17) $$\delta_{kj}=\left\{ \begin{array}{l} 0,k\neq j\\ 1,k=j \end{array}\right.$$

At time $t_{k}$, the estimation ${\hat{X}}_{k}$ of the error state vector $X_{k}$ could be obtained by KF. Thus, the error covariance matrix of the estimation is described as Equation (19)

(18) $$E\left[({\hat{X}}_{k}-X_{k})({\hat{X}}_{k}-X_{k}{)}^{T}\right]\;=\;P_{k}$$

On the diagonal of the error covariance matrix $P_{k}$, it is the mean square estimation error (MSEE) of each error state which represents the estimation accuracy.

The estimation process starts by providing the prediction ${\hat{X}}_{k/k-1}$ of the state vector as follows

(19) $${\hat{X}}_{k/k-1}=\Phi_{k,k-1}{\hat{X}}_{k-1}$$

Secondly, we should predict the value of the error covariance matrix $P_{k/k-1}$

(20) $$P_{k/k-1}=\Phi_{k,k-1}P_{k-1}\Phi_{k,k-1}^{T}+\;\Gamma_{k-1}Q_{k-1}\Gamma_{k-1}^{T}$$

Thirdly, with the error covariance matrix $P_{k/k-1}$, the Kalman gain matrix $K_{k}$ is computed by Equation (21).

(21) $$K_{k}=P_{k/k-1}H_{k}^{T}{\left(H_{k}P_{k/k-1}H_{k}^{T}+R_{k}\right)}^{-1}$$

Next, the estimation ${\hat{X}}_{k}$ could be obtained as follows:

(22) $${\hat{X}}_{k}={\hat{X}}_{k/k-1}\;+\;K_{k}(Z_{k}-H_{k}{\hat{X}}_{k/k-1})$$

Finally, the error covariance matrix $P_{k}$ of the estimate ${\hat{X}}_{k}$ could be calculated by the following equation:

(23) $$P_{k}=(I-K_{k}H_{k})P_{k/k-1}$$

Based on the above equations, it can be seen that the Kalman gain matrix $K_{k}$ is the major contributor to MSEE. $K_{k}$ is directly proportional to the estimate error covariance $P_{k/k-1}$ and inversely proportional to the variance of the measurement noise $R_{k}$. According to Equation (20), $P_{k/k-1}$ is directly proportional to the system noise variance $Q_{k}$. In conclusion, the $K_{k}$ is also proportional to the system noise $Q_{k}$. Therefore, the Kalman gain $K_{k}$ presents a ratio of the uncertainty in the state estimate to the uncertainty in observations. Therefore, the accuracy of the measurement noise model $R_{k}$ and the system noise model $Q_{k}$ could influence the estimation results of the KF. Because the measurement information is always the outer information, we don’t know it very accurately. The only way to improve the accuracy of the KF is to establish an accurate system noise model for the inertial sensors.

### 4.2. Noise Model Based on DAVAR

The system noise vector $Q_{k}$ is always assumed to be white noise whose covariance given by the manufacturer or by the datasheet. However, the noise of the inertial sensors isn’t simple white noise. Especially in the vibration, it contains a lot of color noise. For the sake of providing an optimal estimation of the error states as mentioned above, we established a precise model for the sensors using the DAVAR. On the basis of Equation (6), the variance of the noise at each time in the *X* axis gyro, *Y* axis gyro and *Z* axis gyro could be obtained. The variance of the noise at time *t* can be expressed as follows:

(24) $$Q_{k}(t)=\left[\begin{array}{cccc} 0_{3\times 3} & 0_{3\times 3} & 0_{3\times 3} & 0_{3\times 6}\\ 0_{3\times 3} & \begin{array}{c} Q_{3\times 3}^{a} \end{array} & 0_{3\times 3} & 0_{3\times 6}\\ 0_{3\times 3} & 0_{3\times 3} & \begin{array}{ccc} \sigma_{\mathrm{gx}}(t) & 0 & 0\\ 0 & \sigma_{\mathrm{gy}}(t) & 0\\ 0 & 0 & \sigma_{\mathrm{gz}}(t) \end{array} & 0_{3\times 6}\\ 0_{6\times 3} & 0_{6\times 3} & 0_{6\times 3} & 0_{6\times 6} \end{array}\right]$$

$Q_{3\times 3}^{a}$ is the constant variance of the noise in the accelerometers which can be got from the manufacture. $\sigma_{\mathrm{gx}}(t)$, $\sigma_{\mathrm{gy}}(t)$ and $\sigma_{\mathrm{gz}}(t)$ is the DAVAR of the *X* axis gyro, *Y* axis gyro and *Z* axis gyro, respectively. Instead of a constant system noise model, the DAVAR noise model is dynamic model which changes with time and varies with different motion. Moreover, it contains not only the white noise, but also the color noise. Applying the DAVAR noise model to the Kalman filter will improve the accurate and performance of the Kalman filter estimation.

## 5. Experiments

**Figure 1:**
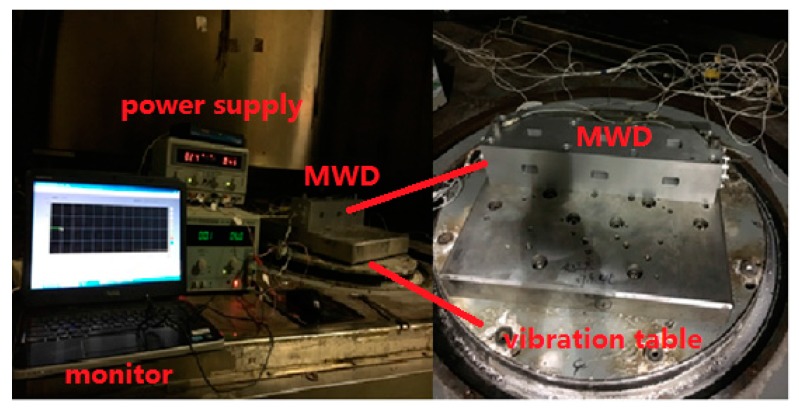
Vibration experiment setup.

**Figure 2:**
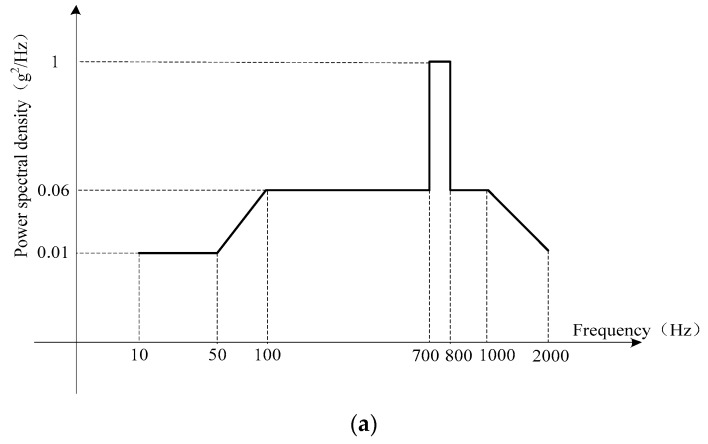

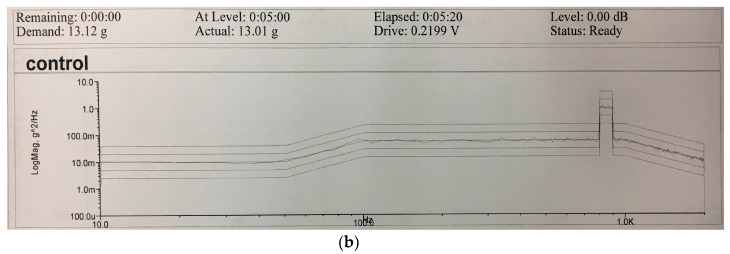
power spectral density (PSD) of the random vibration. (**a**) PSD of the vibration; (**b**) the output of the vibration table.

In order to validate that the proposed noise model based on DAVAR outperforms the conventional one, two vibration experiments were done in the laboratory located in Beijing, China whose latitude is 39.9778° and longitude is 116.3448°. The FOGs-based MWD system was mounted on the horizontal vibration platform as Figure 1 was shown. The vibration table was a 5T vibrating table produced by the American Ling Company, Model 1216VH, No. 219. It is a linear vibration table. The accuracy of the FOG in the MWD under test is 0.5°/h (1σ) and its total length of the optic fiber in the fiber coil is 600 m. The first one was a sine vibration test with a fixed frequency 25 Hz and fixed vibration acceleration 5 g which imitated the low frequency vibration existing in the drilling process. As the Standards of the petroleum and natural gas industry of the People’s Republic of China requires [26], the MWD should perform such a vibration for more than 1 hour to test its reliability before putting the MWD instrument into practice. The second one was random vibration whose vibration PSD was reported in Figure 2. Figure 2a is the demand vibration PSD. The Figure 2b is the output of the vibration table. The Root-mean-square of the whole vibration magnitude is 13.12 g. While drilling, a vibration sensor was mounted on the MWD and collected the vibration information down hole. This vibration PSD was the FFT result of the vibration sensor data. Therefore, it can simulate strong vibration while drilling.

Because the MWD vibrated in the fixed place, its position didn’t change and was always the same as the initial position. Its velocity could be assumed as zeros with random noise. Therefore the aiding source for the vibration experiment is the initial position and the velocity. The measurement vector in Equation (12) can be described as follows;

(25) $$Z_{k}={\left[\begin{array}{cccccc} L_{ins}-\;L_{initial} & \lambda_{ins}-\;\lambda_{initial} & h_{ins}-\;h_{initial} & V_{ins}^{e}-0 & V_{ins}^{n}-0 & V_{ins}^{u} \end{array}-0\;\right]}^{T}$$

### 5.1. Fixed-Frequency Vibration

**Figure 3:**
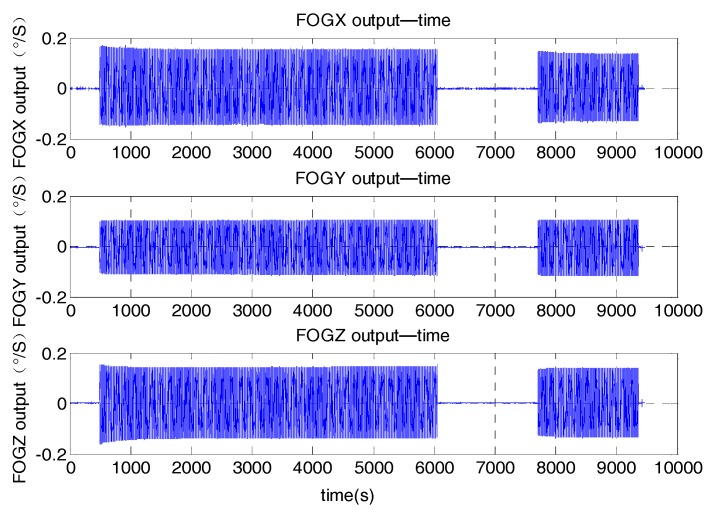
Raw data of the Fiber Optic Gyroscopes (FOGs) in the fixed frequency vibration.

This vibration test was performed as the steps below. First, the vibration table was in static for about 5 min. Then the vibration table started to vibrate. After vibrating for about 1.5 h, the vibration table returned to static. Next, the vibration table started to vibrate again and vibrated for about 15 min. The vibration direction was along the *X* axis of the MWD. This experiment with different motions can motivate the FOG noise. The raw date of the three FOGs was represented in Figure 3. It can be seen that during the vibration, the noise existing in the FOG data became very large. It was different from the noise in the static, so modeling the FOG noise during vibration as the stationary white noise was not corrected.

Then the noise modeling approach based on DAVAR has been used to analyze the vibration data. Its results are obtained with a truncation window whose length is $N_{W}=50,000$ samples, and step width is 5000 samples.

**Figure 4:**
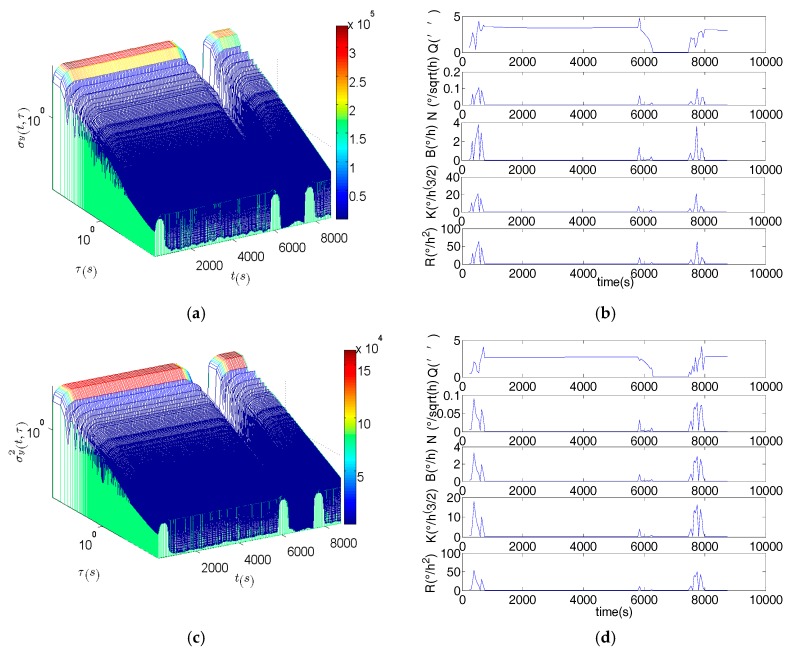

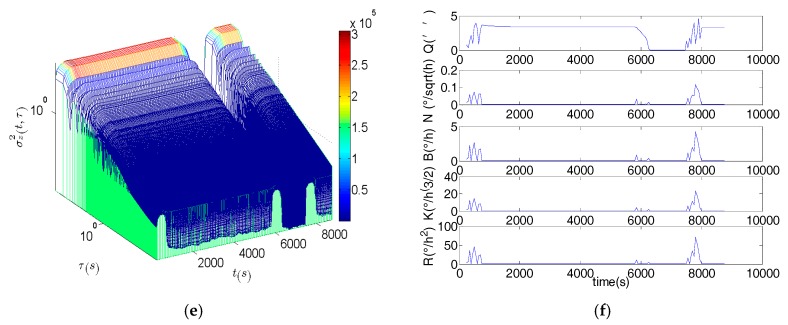
Dynamic Allan variance (DAVAR) and the noise term of the fixed-frequency vibration test. (**a**) The DAVAR of the FOG along *X* axis; (**b**) The coefficients of noise terms in the FOG along *X* axis; (**c**) The DAVAR of the FOG along *Y* axis; (**d**) The coefficients of noise terms in the FOG along *Y* axis; (**e**) The DAVAR of the FOG along *Z* axis; (**f**) The coefficients of noise terms in the FOG along *Z* axis.

**Figure 5:**
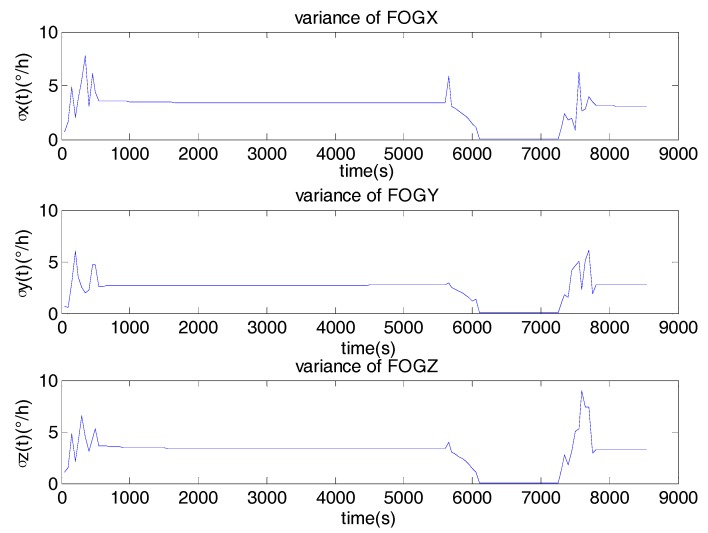
The noise model based on DAVAR.

Figure 4 is the DAVAR results. The surface of the DAVAR is stationary both in the static and in vibration. But when the motion status of the FOG is changing, the surface of the DAVAR appears to have big crests which can be seen clearly at *t* = 300 s, *t* = 6000 s and 7800s. The DAVAR results are consistent with the raw data of the FOGs in Figure 3. Fitting the bi-logarithm graph $\sigma^{2}(t,\tau )-\tau$ at each analysis time *t*, the coefficients of each noise terms can be acquired as Figure 4b,d,f is showing. At time points *t* = 300 s, *t* = 6000 s and 7800s, all the noise terms are very large. During the vibration (*t* = 300 s to 6000 s), the magnitude of the noise coefficients is as small as that in the static, except for the Quantization noise. Though the Quantization noise is larger, its value doesn’t change greatly in vibration. So we can conclude that the noise terms excepting Quantization noise were not motivated by the fixed frequency vibration. The reason is that the fixed-frequency vibration is a stable and disciplinary motion without extreme changing. The Quantization noise has been motivated because any dynamic motion will make the Quantization error larger than that in the stationary. But when MWD began to vibrate and when it was back to static, all the noise items of MWD became bigger. Therefore, the DAVAR could track and reveal the instability of the noise items in the FOGs data. With the DAVAR results, we can establish a precise model for the vibration noise. The noise model based on DAVAR is showed in Figure 5.

Figure 5 shows the variance of FOGs noise derived from DAVAR. It can be seen clearly that the noise in different motions is different. While the motion of the gyro is changing from static to vibration, the variance of the noise is the largest in the whole experiment. During the vibration, the variance of the noise is bigger than that in the static. Table 2 lists the variance of the noise at different time epochs. In static, the noise is the smallest. In vibration, the noise becomes much bigger. When the state of the motion is changing, the noise in the FOG is the biggest. Therefore, In the KF, if the system noise is modeled as a stationary white noise with a constant variance, it must result in degrading the performance and the accuracy of the KF.

**Figure 6:**
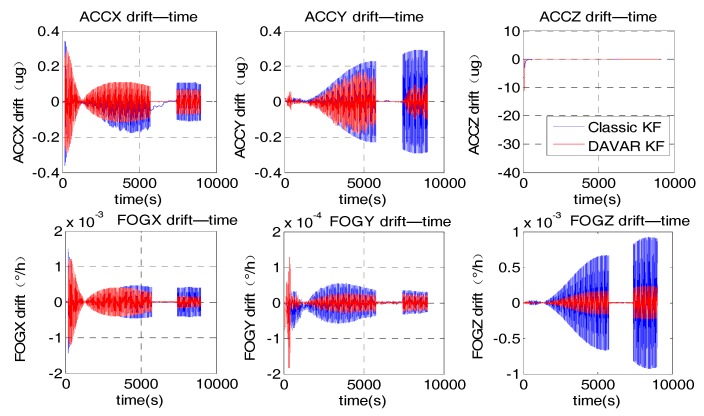
Estimate of FOGs drifts and Accelerometers drifts.

We applied the proposed noise model to the KF, namely DAVAR KF. In order to prove that the proposed noise model based on DAVAR can improve the performance of the KF, it was compared with the classic KF with a white noise model. We labelled it as the classic KF. The variance of the white noise model was set to 0.02°/h since the nominal accuracy of the FOG is 0.02°/h. While the white noise model only includes white noise, the noise model based on DAVAR has taken all the possible stochastic noise in inertial sensors into consideration. Thus, with this new model, the KF can estimate the inertial sensors drifts more accurately. Figure 6 represented the estimate value of FOGs drifts and Accelerometers drifts.

In the Kalman Filtering, the drifts of the inertial sensors (gyros and accelerometers) are estimated and compensated consecutively. That is, in every filtering cycle, the residual drifts are estimated and compensated. Hence, if the KF works normally, after the consecutive compensation, the estimated drifts should converge to zero gradually. In Figure 6, it can be noted that the maximum absolute amplitude of the sensor drifts estimated using the classical noise model is much larger than that using the proposed noise model. With the proposed model, the estimated value for the sensor drifts converged to smaller values which were almost zero, not only for the bias error of the gyros, but also for the bias error of the accelerometers. With this developed method, the whole performance of the KF had become better and the sensor’s drift can be estimated and compensated more accurately with the DAVAR noise modeling approach.

**Figure 7:**
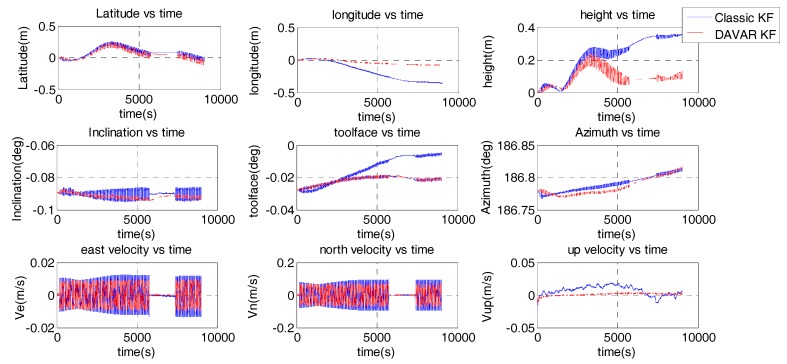
Navigation results of the fixed-frequency vibration.

Before vibration, we have 5 min to alignment. The alignment results are that the inclination angle is −0.089°, toolface angle is −0.028° and the Azimuth is 186.780°. The navigation results based on these two noised models are compared in Figure 7.

Because the MWD didn’t move in this experiment, the position results and velocity results are also the navigation errors. It can be noted that the longitude is 0.42 m using the conventional noise model, while it becomes 0.069 m using the proposed noise model. The longitude error is decreased by 83.6%. The height is also reduced from 0.4 m to 0.13 m. The fluctuation range of the inclination error is repressed by the DAVAR noise model. The toolface error computed by the classic KF is divergent with time. While using the DAVAR KF, it is converged to 0.02°. Because the FOG based MWD is vibrating, the velocity is fluctuating around zero. It can be seen that the fluctuation range of the velocity calculated by DAVAR KF is smaller than the classical KF. Hence, the accuracy of the DAVAR KF is higher than the classic KF.

### 5.2. Random-Frequency Vibration

**Figure 8:**
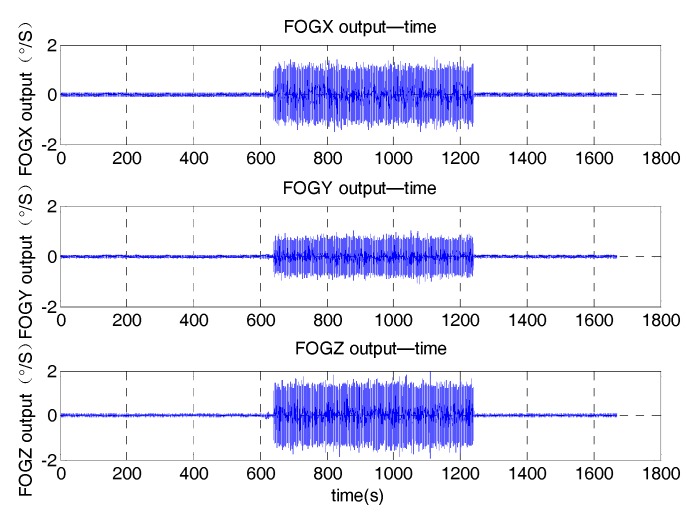
Raw data of the FOGs in the random vibration.

This random vibration test was implemented as the steps below. Firstly, the vibration table was stationary for about 10 min. Then the vibration platform began to vibrate and kept vibrating for about 10 min. Finally the vibration platform returned to static and kept static for about 10 min. The vibration direction of the MWD system was along the *X* axis. The raw data of the three FOGs is represented in Figure 8. During the vibration, the noise existing in the FOG data is very big. The magnitude of the noise is about 2°/s in the vibration.

Then the DAVAR noise modeling approach has been utilized to model this vibration error. The DAVAR was computed by a truncation window of length $N_{W}=50,000$ samples, and a step of width 5000 samples.

**Figure 9:**
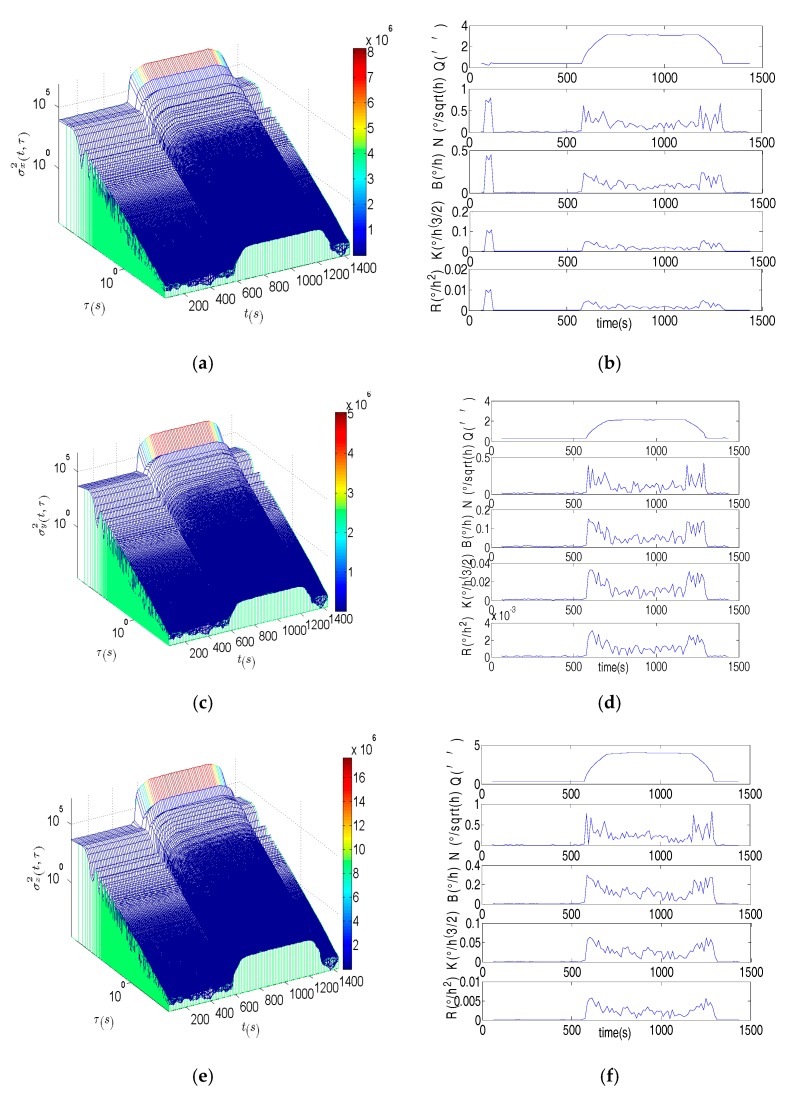
DAVAR and the noise terms in the random vibration test. (**a**) The DAVAR of the FOGX; (**b**) The coefficients of noise terms in FOGX; (**c**) The DAVAR of the FOGY; (**d**) The coefficients of noise terms in FOGY; (**e**) The DAVAR of the FOGZ; (**f**) The coefficients of noise terms in FOGZ.

Figure 9 shows the DAVAR results. DAVAR surface was stationary when the vibration table was static. Then a big crest which started at *t* = 600 s and stopped at *t* = 1200 s was appearing. At the end of the test, the DAVAR came back to the stationary. Fitting the bi-logarithmic curve $\sigma^{2}(t,\tau )-\tau$, the noise items can be obtained as Figure 9b,d,f was shown. Before vibration (*t* < 600 s), each coefficient was small and they didn’t have obvious change. While the vibration table began to vibrate (*t* = 600 s), each noise term changed sharply. During vibration the noise items change obviously, which is different to the noise items in the fixed frequency vibration. After vibration, coefficients of noise terms were all back to the small value, similar to the value in static. In conclusion, the noise in the FOGs is not always unchangeable and the DAVAR could identify and reveal the highly dynamic instability in the FOG’s data.

**Figure 10:**
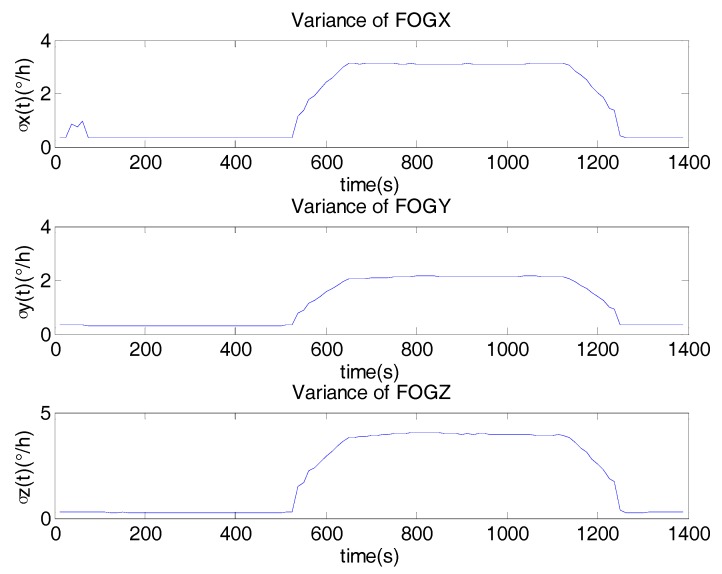
Noise model based on DAVAR.

**Figure 11:**
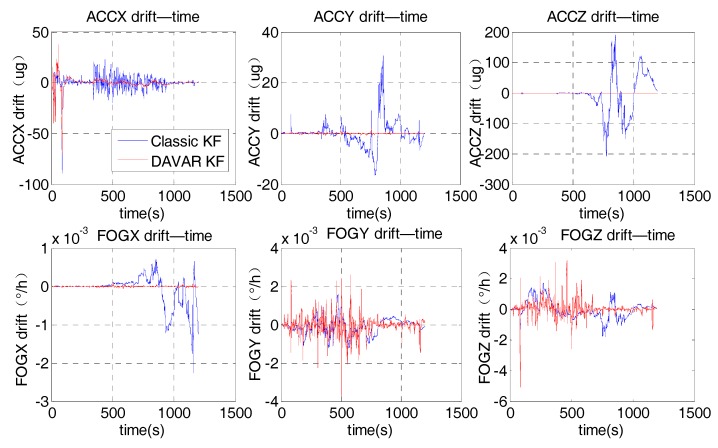
Estimate drifts of FOGs and ACCs.

Based on the Equation (6), the noise model based on DAVAR could be obtained. Figure 10 shows the variance of FOGs noise derived from the DAVAR. It can be seen clearly that the noise in the random vibration is very big. The variance of the noise in the vibration is 4°/h, while it is only 0.03°/h in the static state. Then this proposal noise model was applied to the KF. Figure 11 represents the estimate drifts of the FOGs and ACCs.

Using the proposed model, the maximum absolute amplitude of estimation value of the gyros’ drifts was much smaller than that by the conventional method. Though the fluctuation amplitude for the *Y* axis FOG and *Z* axis FOG is a littler bigger, they converge to a smaller value after 1000 s. It proved that, compared to the classical KF, the sensor’s drift can be estimated and compensated more accurately with the proposed approach.

**Figure 12:**
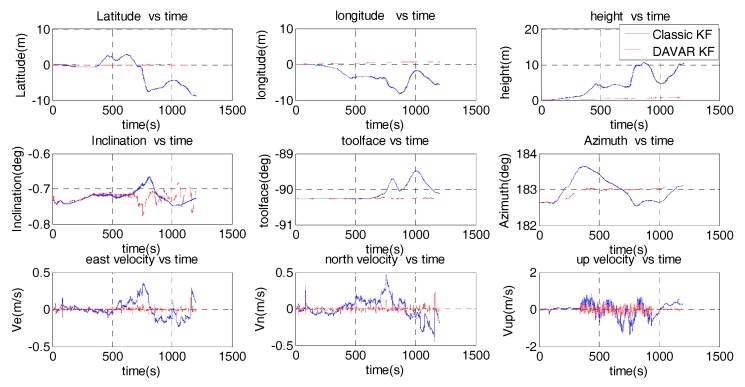
Navigation results of the random vibration.

The navigation results based on the DAVAR noise model and the classical white noise model are reported in Figure 12.

Using the classic KF, the position diverges with time. After 20 min random vibration, the latitude, the longitude and the height are all increased to 10 m. On the contrary, using the DAVAR noise model, the position is limited to 0.27 m, 0.697 m and 0.741 m, respectively. So the position error is reduced more than 90% by the DAVAR KF. Comparing the attitude to the alignment result (inclination angle is −0.735°, the toolface angle is −90.278° and the azimuth is 182.633°), the attitude error from the DAVAR model displays a smaller drift than the classical one. We can see that the toolface angle obtained by the classic KF drifts to −0.78°while the DAVAR KF drifts only 0.02°. Using the classical KF, the maximum fluctuation range of the azimuth is 1°. While using the DAVAR KF, the maximum fluctuation range of the azimuth is 0.5°. For the velocity, when using the classical noise model, the maximum absolute amplitude error of the velocity is 0.5 m/s. However, when using the DAVAR noise model, the maximum fluctuation range of velocity is only 0.2 m/s. Table 3 listed the navigation error obtained from both of the classic KF and the DAVAR KF. It can be seen that each kind of navigation error is decreased dramatically. The position error was reduced more than 90%. The velocity error was reduced more than 60%. The attitude error was reduced by 30% at least. Therefore, the navigation results obtained using the DAVAR noise model are much more accurate than using the conventional one.

## 6. Conclusions

This paper proposed a new noise modeling approach for vibration noise and applied this new noise model to the Kalman filter. Firstly, the random noise items were identified and separated using dynamic Allan variance. Then, the noise model including white noise and color noise was established based on the results of DAVAR. Finally, this new noise model was applied to the Kalman filter to provide an accuracy model for the system noise. Two vibration experiments have been performed to validate the new noise modeling approach. One was with fixed frequency vibration and another one was with random vibration. Both the experiments’ results demonstrated that the proposed approach could improve the performance and accuracy of the KF greatly, especially for the random vibration experiment. For the random vibration experiment, using the DAVAR KF, the position error was reduced more than 90%. The velocity error was reduced more than 60%. The attitude error was reduced by 30% at least.

## Figures and Tables

**Table 1:** Relationship between noise terms, Allan variance and τ.

Noise Terms	Noise Coefficient	$S_{\Omega }(v)$ PSD of the Random Process	$\sigma_{}^{2}(\tau )$	Slope of $\log\sigma_{}^{}(\tau )\;-\;\log\tau$
the quantization noise	*Q*	$S_{\Omega }(f)\;=\;\{\begin{array}{c} \frac{4Q^{2}}{\tau }\sin^{2}(\pi f\tau )\\ {\left(2\pi f\right)}^{2}\tau Q^{2} \end{array}\begin{array}{cc} & f\geq \frac{1}{2\tau }\\ & f<\frac{1}{2\tau } \end{array}$	$\frac{3Q^{2}}{\tau^{2}}$	−he
angular random walk	*N*	$S_{\Omega }(f)\;=\;N^{2}$	$\frac{N^{2}}{\tau }$	−ngul
bias instability	*B*	$S_{\Omega }(f)=\{\begin{array}{c} (\frac{B^{2}}{2\pi })\frac{1}{f}\\ 0 \end{array}\begin{array}{cc} & f\leq f_{0}\\ & f>f_{0} \end{array}\;f_{0}\text{ cutoff frequency}$	$\frac{2B^{2}}{\pi }\ln2$	0
rate random walk	*K*	$S_{\Omega }(f)\;=\;(\frac{K^{2}}{2\pi })\frac{1}{f^{2}}$	$\frac{K^{2}\tau }{3}$	0.5
the rate slope	*R*	$S_{\Omega }(f)\;=\;\frac{R^{2}}{{\left(2\pi f\right)}^{3}}$	$\frac{R^{2}\tau^{2}}{2}$	1

**Table 2:** The variance of the noise.

Motion	FOGX Noise	FOGY Noise	FOGY Noise
During vibration (3000 s)	3.404	2.689	3.379
Static (6500 s)	0.023	0.012	0.009
Static to vibration (6800)	6.248	6.058	8.959

**Table 3:** The navigation error.

Parameter	Classic KF	DAVAR KF	Optimized
Latitude error (m)	8.562	0.257	96.99%
Longitude error (m)	7.962	0.697	91.25%
Height error (m)	10.129	0.741	92.68%
Inclination error (deg)	0.06	0.04	33.33%
toolface errer (deg)	0.78	0.02	97..45%
Azimuth error (deg)	1	0.5	50%
East velocity error (m/s)	0.3415	0.05	85.36%
North velocity error (m/s)	0.452	0.160	64.60%
up velocity error (m/s)	1.191	0.399	66.50%
